# Detection of Self Incompatibility Genotypes in *Prunus africana*: Characterization, Evolution and Spatial Analysis

**DOI:** 10.1371/journal.pone.0155638

**Published:** 2016-06-27

**Authors:** Judith Ssali Nantongo, Gerald Eilu, Thomas Geburek, Silvio Schueler, Heino Konrad

**Affiliations:** 1 National Forestry Resources Research Institute, Mukono, Uganda; 2 Makerere University, Kampala, Uganda; 3 Austrian Federal Office and Research Centre for Forests (BFW), Vienna, Austria; Aristotle University of Thessaloniki, GREECE

## Abstract

In flowering plants, self-incompatibility is an effective genetic mechanism that prevents self-fertilization. Most *Prunus* tree species exhibit a homomorphic gametophytic self-incompatibility (GSI) system, in which the pollen phenotype is encoded by its own haploid genome. To date, no identification of *S*-alleles had been done in *Prunus africana*, the only member of the genus in Africa. To identify *S*-RNase alleles and hence determine *S*-genotypes in African cherry (*Prunus africana*) from Mabira Forest Reserve, Uganda, primers flanking the first and second intron were designed and these amplified two bands in most individuals. PCR bands on agarose indicated 26 and 8 different *S*-alleles for second and first intron respectively. Partial or full sequences were obtained for all these fragments. Comparison with published *S*-RNase data indicated that the amplified products were *S*-RNase alleles with very high interspecies homology despite the high intraspecific variation. Against expectations for a locus under balancing selection, frequency and spatial distribution of the alleles in a study plot was not random. Implications of the results to breeding efforts in the species are discussed, and mating experiments are strongly suggested to finally prove the functionality of SI in *P*. *africana*.

## Introduction

*Prunus africana* (Hook. f) Kalkman is a medicinal tree indigenous to the montane regions of West, Central, East and Southern Africa, including Madagascar. It is the only member of the genus *Prunus* which comprises of more than 200 species that are indigenous to Africa [1] where are found in fragmented populations. The species is economically important in Uganda, Cameroon, Madagascar and Kenya for its bark extract that is used to treat benign prostatic hypertrophy. However, unsustainable harvesting has threatened the survival of the tree and it is currently classified as an endangered species by the Convention for International Trade in Endangered Species (CITES) [2]

Taking into account the economic importance of *P*. *africana*, a number of *in-situ* and *ex-situ* initiatives aimed at sustainably conserving and managing the species have been initiated. However, long term sustainability of *ex-situ* initiatives may be constrained if the species is self-incompatible as are many other *Prunus* species. Most of these species operate a strictly homomorphic gametophytic self-incompatibility (GSI) system, in which specificity of self/non self-recognition is controlled by products encoded within the *S* -locus. Self incompatibility may be a hindrance to breeding for pure lines and the distribution and frequency of *S*-alleles may affect mating success, and consequently gene dispersal patterns, spatial genetic structure and genetic diversity [3]. Compatible crosses require distinct alleles so that the population-level rare alleles are favored. On the other hand, SI is of evolutionary importance in flowering plants due to its effectiveness in avoiding inbreeding and encouraging outcrossing which helps to promote heterozygosity and fitness [4].

*S*-RNase genes have been identified and characterized in several *Prunus* species including *Prunus dulcis*, *P*. *avium*, *P*.*cerasus and P*. *mume* [5–8]. Characterization of *S*-RNases in Rosaceae shows that there can be in excess of thirty different *S*-alleles. The aim of this work was to identify and characterize the *S*-RNase gene associated with gametophytic SI in *P*. *africana*. The results will be of use in breeding and seed production of *P*. *africana* during selection of individuals for cross-fertilization.

## Materials and Methods

### Study area and Population description

Leaf samples were collected from Mabira forest reserve, located in South Central Uganda between 0o 22’–0o 35’N and 32o56’–33o 02’E. It is a mid-elevation forest located between 1070 and 1340 m a.s.l, with a total area of 300 km², occupying gently undulating plains with numerous flat-topped hills and wide shallow valleys. About 95% of the area is covered by medium altitude moist semi-deciduous forest [9]. The remaining portion is occupied by medium altitude moist evergreen forest. The three recognized sub-climaxes are: colonizing forest, mature mixed forest and *Celtis* mixed forest. Although some parts of the forest are degraded, there is still an undisturbed nature reserve.

**Fig 1:**
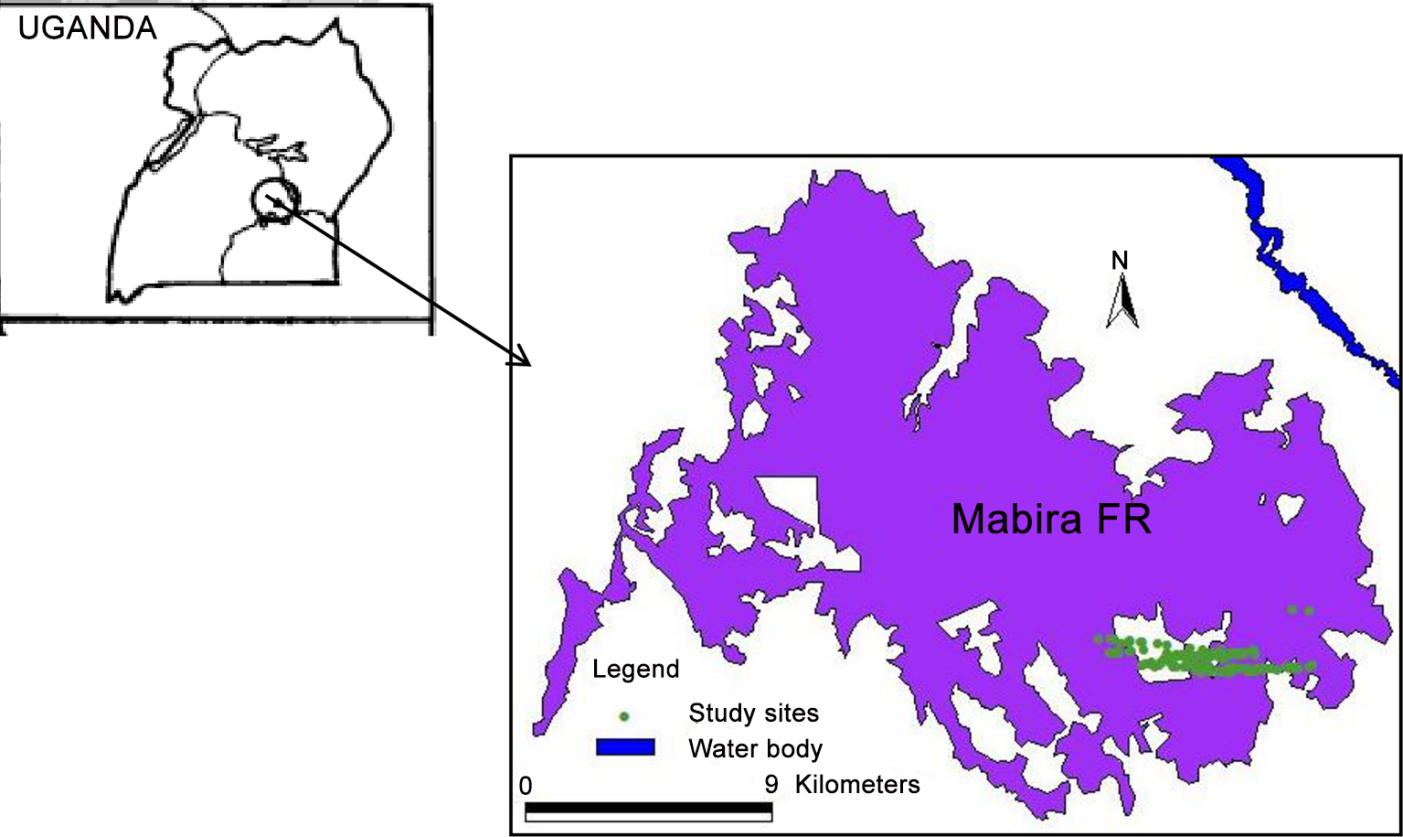
Map showing the location of Mabira forest and the individual trees of *P*. *africana* sampled for S-allele analysis

The initial reconnaissance survey of *P*. *africana* in Mabira forest showed that it occurs in demes separated by a minimum of 30 m (Fig 1). The isolated demes have a minimum number of 50 mature trees. Samples for this study were collected from the intensively studied plot in the more intact nature reserve. A total of 150 trees were sampled.

### Sample Collection and DNA extraction

From the intensively studied plot, young and disease free leaf samples of 150 trees were collected. The leaves were then cleaned with absolute ethanol and then sliced into small pieces before they were put into zip lock bags filled with self indicating blue silica gel. The individual tree locations were geo-referenced with a Global Positioning System (GPS) for easy monitoring and for spatial genetic structure analysis (Table 1). The leaf samples were shipped to the Federal Research and Training Centre for Forests, Natural Hazards and Landscape (BFW) genetic laboratory for DNA extraction and analysis. Total genomic DNA was extracted from about 10g of all the leaf samples following documented protocols [10]. The quality and purity of genomic DNA was evaluated by a ND-1000 spectrophotometer (NanoDrop, USA)) and by gel electrophoresis. For gel electrophoresis, 1 μl of each DNA extract was analysed in a 1.5% agarose gel containing 0.5% ethidium bromide and was visualized by U.V. illumination.

**Table 1:** GPS coordinates of all the *Prunus africana* trees that were sampled for analysis of S-alleles.

TREE NO	LONGITUDE	LATITUDE
1	50.165	0.42677
2	50.1633	0.42678
3	50.1643	0.42685
4	50.1641	0.42664
5	50.1627	0.42664
6	50.1584	0.42627
7	50.1528	0.42599
8	50.1472	0.4246
9	50.1519	0.42578
10	50.1438	0.42507
11	50.1551	0.42471
13	50.1511	0.42388
15	50.1571	0.42392
16	50.1593	0.42412
17	50.159	0.42395
19	50.1668	0.42331
20	50.1658	0.42313
21	50.1673	0.42317
22	50.1704	0.42323
23	50.1747	0.42313
24	50.1754	0.42308
25	50.1785	0.42305
26	50.1796	0.4231
27	50.1788	0.42322
28	50.177	0.42343
29	50.1764	0.42334
30	50.176	0.42332
31	50.1716	0.42352
32	50.1712	0.42342
33	50.1695	0.42343
34	50.1679	0.42343
35	50.168	0.42342
36	50.1673	0.42345
37	50.1667	0.42368
38	50.1637	0.42644
41	50.1608	0.42298
42	50.1815	0.4227
43	50.1552	0.42278
44	50.1596	0.42209
45	50.1569	0.42155
46	50.1657	0.42173
47	50.1619	0.42217
48	50.1636	0.42224
49	50.1669	0.42241
50	50.1736	0.4227
52	50.1771	0.42264
53	50.1746	0.42244
54	50.1737	0.42236
55	50.1734	0.42253
56	50.1721	0.4225
59	50.1858	0.42324
60	50.1875	0.42313
61	50.1869	0.42308
62	50.1888	0.42318
65	50.1857	0.42329
66	50.1862	0.42329
69	50.1875	0.42366
70	50.1862	0.42376
71	50.1863	0.44238
72	50.1923	0.42398
73	50.1921	0.44215
74	50.1931	0.42403
78	50.1847	0.42368
83	50.1469	0.4249
84	50.1405	0.4252
85	50.1403	0.4246
86	50.1405	0.42482
87	50.1435	0.42507
88	50.1401	0.42458
89	50.1385	0.42456
90	50.1374	0.42433
91	50.143	0.42405
92	50.1416	0.4242
94	50.1413	0.42364
95	50.1475	0.42368
96	50.1509	0.42362
97	50.1523	0.42395
98	50.1487	0.42385
99	50.138	0.42917
100	50.1678	0.42781
101	50.1682	0.42763
2A	50.1676	0.42783
3A	50.1681	0.42812
4A	50.1695	0.42801
5A	50.168	0.42788
6A	50.1705	0.42787
7A	50.1701	0.42782
8A	50.1745	0.42799
10A	50.1699	0.42818
11A	50.1685	0.42817
12A	50.1714	0.42819
14A	50.1687	0.42804
15A	50.1667	0.42811
17A	50.1634	0.42944
23A	50.1427	0.43112
25A	50.1455	0.43104
27A	50.1532	0.42984
28A	50.1573	0.42938
29A	50.1732	0.42873
31A	50.174	0.42922
34A	50.1633	0.42802
35A	50.1608	0.42793
36A	50.1608	0.4279
37A	50.1592	0.42767
38A	50.1593	0.42755
39A	50.1604	0.42763
41A	50.1598	0.42749
42A	50.1624	0.42765
43A	50.16	0.42762
44A	50.1601	0.42743
46A	50.1522	0.42735
47A	50.1511	0.42736
48A	50.1506	0.4272
49A	50.1515	0.42726
50A	50.1453	0.42809
51A	50.1481	0.42675
52A	50.1472	0.4266
53A	50.1443	0.42646
54A	50.1462	0.4264
55A	50.1493	0.42751
56A	50.1506	0.42764
57A	50.1511	0.42766
58A	50.1504	0.42795
59A	50.1508	0.42779
61A	50.153	0.42767
62A	50.153	0.42761
63A	50.1548	0.42754
64A	50.1563	0.42767
66A	50.1583	0.42811
67A	50.1453	0.42792
68A	50.1483	0.42774
69A	50.1232	0.43271
71A	50.166	0.42758
72A	50.1607	0.42844
73A	50.1608	0.42843
74A	50.1273	0.433
75A	50.1283	0.43295
76A	50.1374	0.43195
77A	50.1368	0.43206
78A	50.1328	0.43163
79A	50.1328	0.4317
80A	50.1305	0.43159
81A	50.1332	0.43167
82A	50.1332	0.43249
100A	50.1337	0.42864
101A	50.1334	0.42867
102A	50.1268	0.42822
103A	50.1278	0.42825
104A	50.1288	0.4282
105A	50.1297	0.42819

### PCR amplification for initial screening

Various consensus polymerase chain reaction (PCR) primers that have been used for identifying *S*-alleles in other *Prunus* species [6][11][12] were tested on 20 samples. The amplicons were purified, sequenced and the results used to design specific primers that were used in this study. Two specific primer pairs (Pacons2F/PAI2-2R and PaI2-2F/PaI2-2R,) flanked the second intron of the S-RNase and three pairs (PaCons1F/PaCons1R, PaSI1f/PaSI1R and Paintron1F/Paintron1R) [12] were used to amplify the first intron. To amplify the first intron, reactions were carried out in a reaction volume of 25 μL containing PCR buffer, 0.4 mM dNTPs, 0.3 μM of each primer and 1.25 U of TaqDNA polymerase (Peqlab). The thermal cycling conditions were similar to those used in documented protocols [12] except that the annealing temperature was raised to 59.8°C. The PCR products were separated on 1.5% agarose gel with the help of Ethidium Bromide (EtBr) staining and the gel image was captured using gel documentation system (Bio-rad).

The second intron was initially amplified using a pair of documented primers [11]. Amplification was carried out in a 25 μL volume containing PCR buffer, 2.5 mM MgCl2, 0.3 mM dNTPs, 1.25 U Taq polymerase and 0.3 μM of each primer. Apart from the annealing temperature which was changed from 58.5°C to 49.3°C the other thermal cycling conditions remained similar to the documented protocols [11]. The extension time was also increased by 12 seconds per cycle. The PCR products were separated on 1.5% agarose gel at 100 V for 30 minutes after ethidium bromide staining. The gel image was taken using Bio-Rad gel documentation system.

### Cloning, sequencing and primer design

For amplifications containing two alleles (most of the cases), bands were cut from the gel, purified and subsequently cloned via a pGEM vector (Fermentas) into *Escherichia coli* competent cells using a heat shock protocol [13] After visually selecting out the cells that were successfully transfected, 7 plasmids per allele were sequenced using universal M13 primers. PCR products of the expected size were purified using the QIAprep Spin Miniprep Kit (Qiagen, Hilden, Germany). In some instances, depending on the length of the insert, internal primers designed from the partial sequences were also used. Specific primers for *P*. *africana* were designed based on the sequences obtained (Table 2).

**Table 2:** Specific primers developed for amplification of S-alleles in *P*. *africana*. Pattern indicates primer combinations.

Primer Pairs	Primer name	Sequence (5’-3’)	Amplified region
Pair 1	Pacons2F	ggccaagtaattattcaaacc	2nd Intron
	PAI2-2R	ttcgccttcccaaaayyttg	2nd Intron
Pair 2	PaI2-2F	ggccaagtaaytattcaaaccca	2nd Intron
	PaI2-2R	ttcgccttcccaaaayyttg	2nd Intron
Pair 3	PaCons1F	mcttgttcttgstttygctttcttc	1st Intron
	PaCons1R	catgratggtgaartwttgtaatgg	1st Intron
Pair 4	PaSI1f	gttcttggtttygctttcttc	1st Intron
	PaSI1R	catgratggtgaagtwttgtaatgg	1st Intron
Pair 5	Paintron1F	ttatgagcastggtgggttg	1st Intron
	Paintron1R	actgggcatckttgggtttg	1st Intron

### Data Analysis

Different size fragments as estimated on agarose were manually scored and distinct allele combinations determined the different genotypes. The fit of the expected and observed distribution was tested using χ2 –test. Mate availability was estimated as percentage of compatible alleles from the observed genotypes [14].

“Sequences” of 7 other Prunus species were obtained from GenBank (http://www.ncbi.nlm.nih.gov). Homologous DNA sequences were aligned using ClustalX [15] and manually adjusted when necessary. Regions that aligned poorly were excluded from the data set. To get a perfect comparison of *P*. *africana* S-RNases with other Rosaceous *S*-RNases, a phylogenetic analysis that grouped *S*-RNases [16] was used. The minimum evolution method for inferring phylogeny using distance matrices with pair wise deletion obtained following the number of differences, as implemented in MEGA software [17] was used. A close-neighbour-interchange heuristic search (level 2) was performed. For the heuristic search, the initial tree was obtained using the distance based Neighbour-Joining method [17]-[18]. The reliability of clustering patterns of the phylogenetic trees was tested by bootstrapping with 1000 pseudo-replicates [19]. Spatial Genetic Structure (SGS) in the population was analyzed with the program SPAGeDi [20]. Closely related pairs of alleles that occupied paired terminal branches on the species-level phylogeny were chosen for analysis of synonymous and non-synonymous substitution rates. Numbers of synonymous substitutions per synonymous sites (Ks) and non-synonymous substitutions per non-synonymous sites (Ka) were calculated using DnaSP [21].

## Results

### Statement of Ethics

Authorization for research within the forest was granted by National Forestry Authority, the managers of Uganda’s Central Forest Reserves. Phytosanitary clearance for the leaf samples was given by the crop protection division of the Ministry of Agriculture, Animal Industries and Fisheries of the government of Uganda.

### *S*-RNase intron polymorphism and genotype frequencies

A total of 142 out of 150 samples successfully amplified using specifically designed primers. Primers developed [6][11],[22]for other *Prunus* species did not effectively amplify the *S*-alleles in most samples indicating that there are pronounced differences in the loci under investigation between *P*. *africana* from other *Prunus* species. As expected of individuals with functional gametophytic SI systems, the alleles were highly polymorphic (Table 3). Of the 142 individuals that were assessed, 98% were heterozygous at the *S*-locus. However, the dual bands of first intron could not be distinctively separated on agarose gel, implying that the two fragments had very small nucleotide differences.

**Table 3:** List of the 26 alleles detected in the second intron of the S-RNase of *P*. *africana*.

Allele No.	Product Size	Count
S1	230	1
S2	250	2
S3	270	22
S4	300	20
S5	400	4
S6	480	7
S7	500	23
S8	510	35
S9	550	4
S10	600	15
S11	650	2
S12	700	13
S13	800	8
S14	900	3
S15	950	2
S16	980	18
S17	1000	20
S18	1200	6
S19	1350	18
S20	1500	2
S21	1700	13
S22	1800	11
S23	2000	1
S24	2500	15
S25	2800	4
S26	3000	15

**Fig 2:**
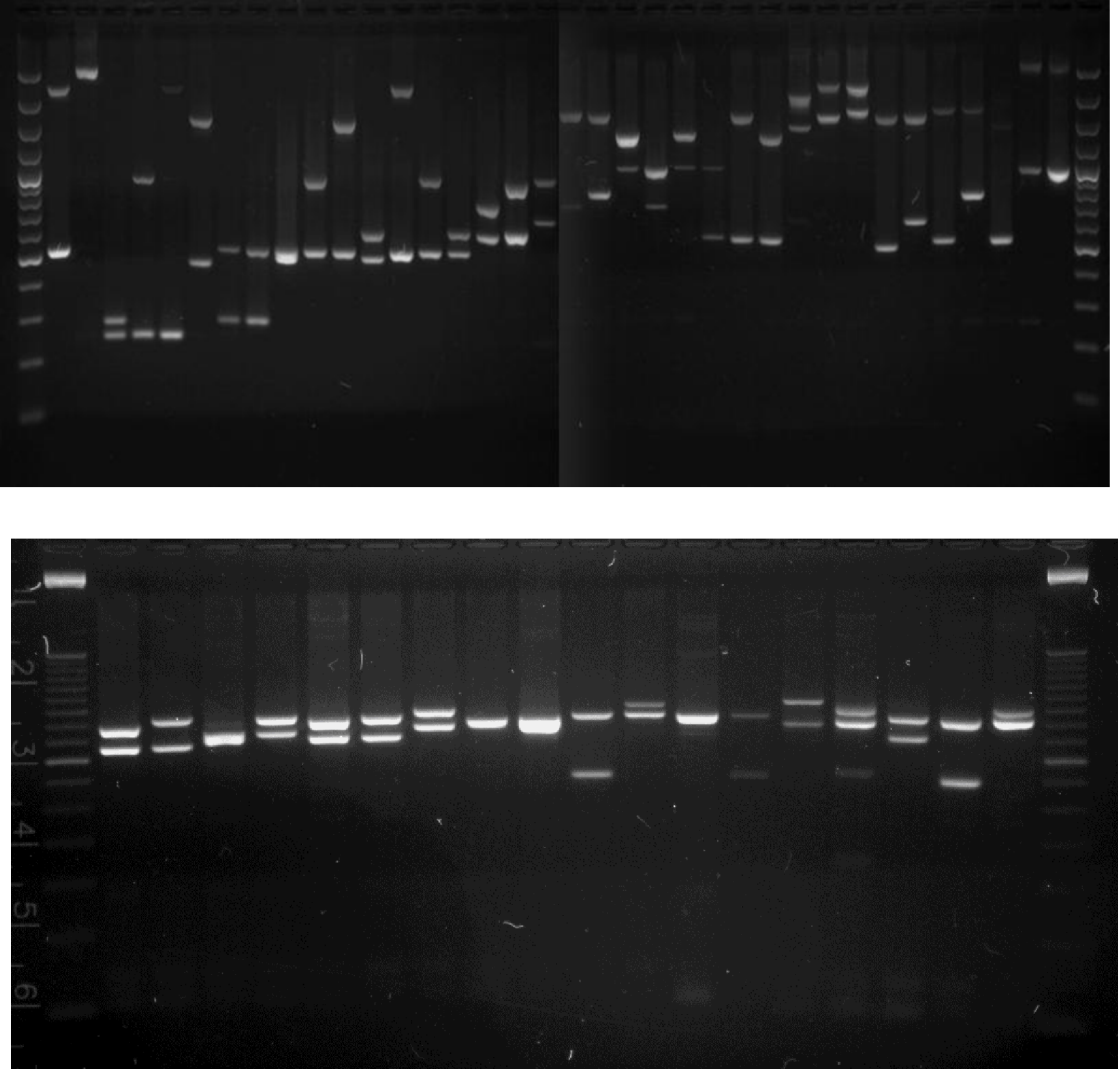
(a) Agarose gel showing amplification products of second intron sequences. First and last lane is the 100 bp size standard. (b) Agarose gel showing amplification products of first intron sequences. First and last lane is the 50 bp size standard

**Fig 3:**
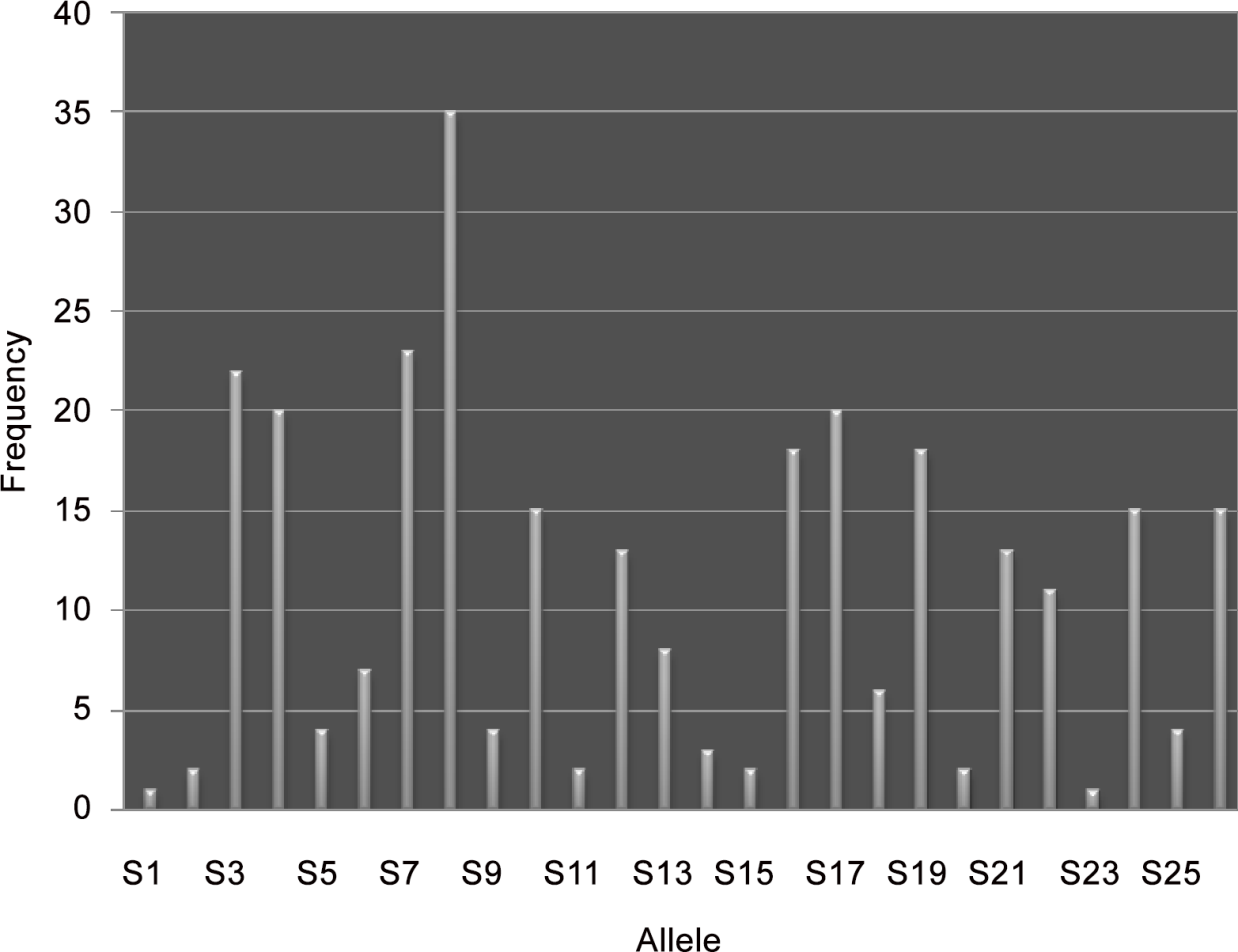
Graph indicating the frequencies of different S-alleles in *P*. *africana*

PCR fragments ranged from 210 bp to 3000 bp. Fragments from the first intron ranged from 210 to 550 bp (Fig 2B) whilst second intron lengths ranged from 245 to 3000 bp (Fig 2A). While 26 different alleles were scored for the second intron, only 8 alleles were identified on the agarose gels for the first intron. The overall *S*-allele frequencies from the second intron are given in Fig 3 and their approximate sizes as scored both on agarose gels and as sequenced product in Table 2 above. The most common allele was S8 (12.3%), while S1 and S23 were least represented (0.3% each). Although, the alleles were expected to be represented in equal frequency under frequency-dependent selection, this was not the case for alleles from both introns as indicated by the Mantel statistic (χ2 = 11.5; P < 0.05). In total, the allele patterns resulted in 54 different genotypes (Fig 2). In this *Prunus africana* population, mating availability ranged from 85% to 100% given the large number of alleles.

### Sequence analysis

Verified using BLAST searches (http://blast.ncbi.nlm.nih.gov), all sequences were from the signal peptide or C1–C5 regions. Several *P*. *africana S*-RNase sequences were very similar to other *Prunus* sequences, both on the intra- and interspecific level. 35 sequences of *P*. *africana* were similar to published sequences of *P*. *Speciosa*, *P*. *dulcis*, *P*. *avium*, and *P*. *Salicina*. Others were similar to alleles found in *P*. *pseudocerasus*, *P*. *webbii*, *P*. *mume*, *P*. *argentea*, *P*. *ceracifera* and *P*. *cerasus*. Although sequences especially those from the second intron could not be reasonably aligned due to their large variation in length, comparison of these sequences with ClustalX [15] showed that there were 40 different sequences. All of these *S*-RNase alleles had a considerably high homology within the coding region and displayed an intron/exon structure characteristic of *Prunus* ribonucleases that is, they contained the five conserved regions, C1, C2, C3, RC4 and C5, as well as the hyper-variable region, RHV, associated with the deduced amino acid sequence of rosaceous *S*-RNases [5].

The regions of the gene that were compared have highly diversified sequences that contained different amino acids ranging from 1 to 10. Pair wise similarity comparison of exons at the nucleotide sequence level showed minimum identity of 50% to maximum identity of 87%, whilst identity at amino acid level was between 20% and 50%. Most identical sequences were generated from more than two representatives of each haplotype. Microsatellites (tandem repeats) mainly AT, GAA, TA were found in several sequences from both introns. Tajiman’s test of neutrality was estimated at 6.2. Distinct sequences were deposited in the GenBank (http://www.ncbi.nlm.nih.gov/) with accession numbers from KT985615 to KT985636.

### Identification of *S*-RNase regions exposed to selection

Closely related pairs of alleles were chosen for analysis of synonymous and non-synonymous substitution rates. Analysis focused on sister alleles that occupied paired terminal branches on the species-level phylogeny. The overall mean values for Ka, Ks and Ka/Ks were 0.1129, 0.096 and 1.18 respectively. An excess of non-synonymous substitutions over synonymous substitutions occurred. The matrix of amino acid differences indicates that the degree of sequence divergence within species is on the same order as the sequence divergence between species. In fact, some alleles appear to be more similar to alleles in other species than to other alleles in the same species.

### Phylogenetic tree analysis

**Fig 4:**
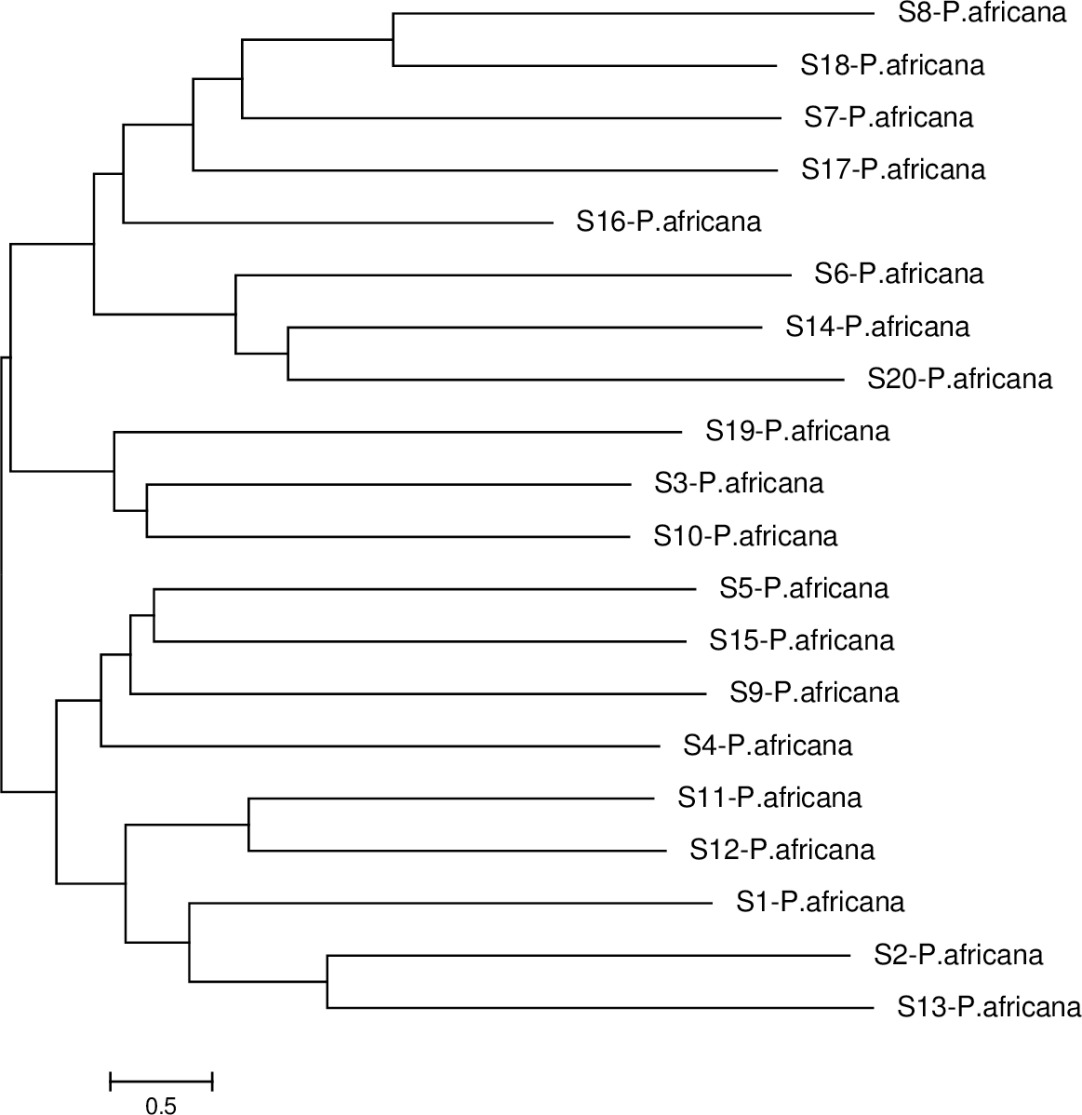
Phylogenetic (minimum evolution) tree of *P*. *africana S*-RNase intron 2 sequences, showing the relationship among alleles.

**Fig 5:**
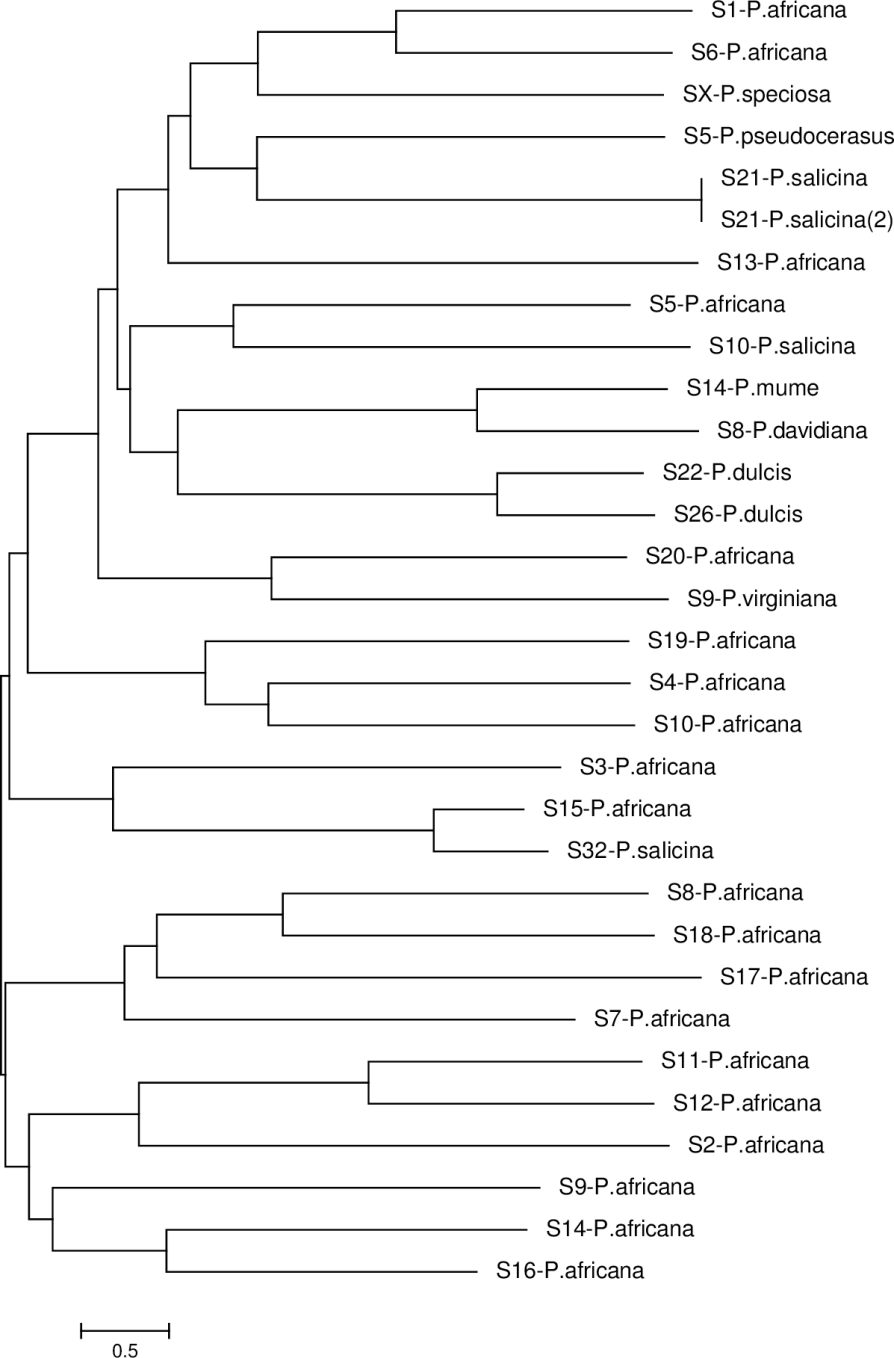
Phylogenetic tree (minimum evolution) of *P*. *africana* and other *Prunus* second intron *S*-RNase sequences depicting relationships with other congeneric species. Allele sequences are highly diverged and partially are similar to *S*-allele sequences obtained in other *Prunus* species, a pattern commonly observed as alleles are older than the species radiation.

Within population *S*-RNase sequences for *P*. *africana* did not show any distinct clusters (Fig 4). However, to place the new *S*-RNase alleles in context with previously identified *Prunus S*-RNase alleles, a phylogenetic tree was constructed comparing *S*-RNases from a representative range of *Prunus* species (Fig 5). Some sequences were excluded after failure to find a reliable root position because of the large genetic distances between alleles from some *Prunus* species. Generally, *S*-RNases *of P*. *africana* often displayed closer relationships to those of other *Prunus* species. This is consistent with trans-specific *S*-allele evolution within *Prunus* as has previously been pointed out [5]. Trans-specific polymorphism involving the new alleles was evidenced by high bootstrap support for grouping of for example S-5 of *P*.*africana* and S-10 of *P*. *salicina*. No specific subgroups within *Prunus* were detected although some pairs of alleles showed high percentages of pair wise amino acid similarity, e.g. *Prunus pseudocerasus* and S-5 of *P*.*africana* (92%). There are also a few monospecific sister pairs while some alleles showed monophyletic clades. Several close pairs of alleles even seem to represent divergent copies of the same specificity yet quite a few alleles did not cluster with any other known *Prunus* alleles.

### Spatial Genetic Structure (SGS)

**Fig 6:**
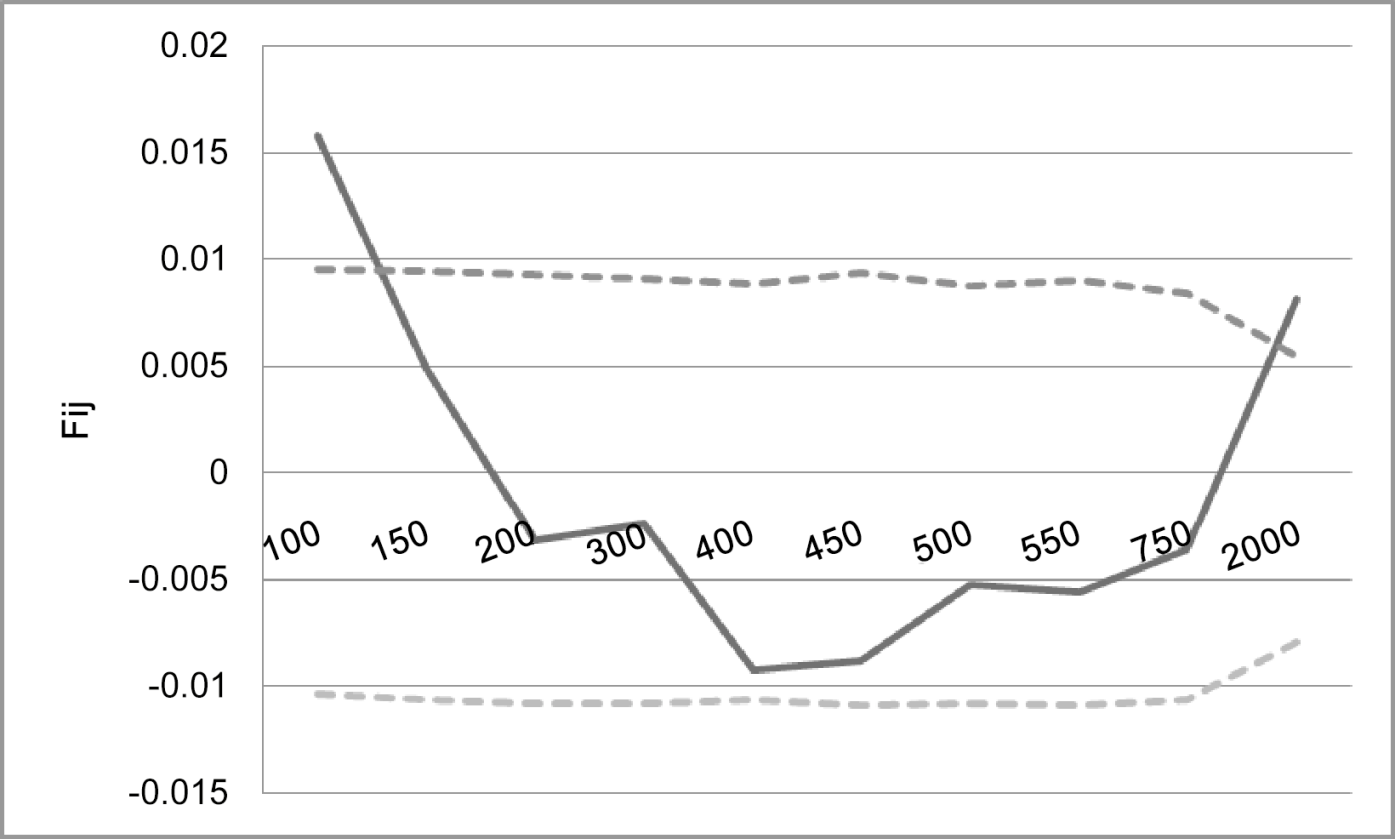
Results of spatial genetic structure analysis in the second intron *S*-alleles of *P*. *africana*. Significant autocorrelation was detected in the first distance class (up to 100 m) between individuals. X-axis is spatial distance between individuals in metres, y-axis is Loiselle’s kinship coefficient for the second intron *S*-alleles

Based on the second intron, the results of fine scale spatial genetic structure (SGS) analysis calculated as correlation between Loiselle‘s kinship coefficient [23] and spatial distance between individuals with 95% confidence limits derived from bootstraps are depicted in Fig 6. Significant SGS was detected only in the first distance class (up to 100 m). No significant SGS was observed in longer distances.

## Discussion

### S-RNase polymorphism and genotype frequencies

The identified *S*-alleles in *P*. *africana* were highly divergent both in length and sequence variation. The number of alleles did not deviate from those observed in other wild and domesticated species of the Rosaceae family which ranges from 12 to 45 [24]. Hoebee [25] for example identified a total of 26 different S-alleles from 138 individuals of *Pyrus pyraster*. A total of 22 alleles were identified in *Prunus lannesiana var*. *speciosa* [26].Twenty alleles were retrieved from 20 *Sorbus aucuparia* individuals, whereas 16, 17 and 22 alleles were found in *Prunus avium* [27], *Crataegus monogyna* [28] and *Malus domestica* [29] respectively. All these above Rosaceae species being diploid, a similar number of specificities was also found in the polyploidy *Prunus spinosa* [16]. The total number of alleles observed in these species represents a higher than expected *S*-allele diversity based on theoretical expectations for gametophytic self-incompatibility system as predicted by the balanced model [30]. For GSI systems, the model stated that “under a moderate mutation/immigration rate of 10^−3^, a population of 50 individuals should harbour about eight different *S*-alleles, whereas a population of 200 individuals would have about 15 *S*-alleles” [30]. However, it is a common occurrence that the number of *S*-alleles found within populations is usually greater than that predicted by theory [24]. A high *S*-allele diversity is often indicative of high immigration rate of new *S*-alleles, which would subsequently experience positive frequency dependent selection [31].

Correspondingly, the mate availability was also expectedly as high as in other Rosaceae species e.g. in *Pyrus pyraster* [32]. This is in agreement with theoretical expectations on mate availability in small populations of plants with a gametophytic self-incompatibility system. Theoretically, a functional gametophytic self-incompatibility (SI) system in plants should exhibit high polymorphism at the SI controlling *S*-locus because individuals with rare alleles have a higher probability to successfully pollinate other plants than individuals with more frequent alleles [3] [32]. Pollen carrying a rare allele will not be rejected by incompatibility reactions of recipient plants and therefore will have higher reproductive success and consequently increased frequency of rare *S*-alleles in subsequent generations. In other words, opportunities for mating of each *S*-allele are inversely related to the alleles' frequencies [30]. Based on these theories, Only populations harboring less than 5 different *S*-alleles should show reduced mate availabilities below 90% [33]. This negative-frequency-dependent selection is explicitly expected to maintain many *S*-alleles in effectively large populations. The very long evolutionary time detected in phylogenetic analyses of *P*. *africana* [34] has also allowed for inclusion of many mutations and new migratory alleles to increase the observed diversity.

Unequal specificity frequencies were observed in *P*. *africana*, although most species with gametophytic SI support the equal allelic frequencies (isoplethy) hypothesis [27], [31] e.g. isoplethy was observed in *Prunus avium* [27]. However, earlier results indicated no departure from the identical allelic frequencies hypothesis in 16 out of 19 sampled populations from 12 species with gametophytic SI [24]. The deviation from isoplethy as of *Prunus africana* may have been caused by the fragmentation of populations as a result of forest degradation, death from overharvesting of the species for bark and the species distribution irregularities. *P*. *africana* is thus found as isolated demes which as a consequence may have suffered different founder events resulting in differentiation at the *S*-locus. Limited dispersal and pollen flow as well as plant size variation that often follow degradation can cause deviation from the expected frequencies [35].

However, it is also urged that it is almost impossible to find equal frequencies when a large sample size is used in analysis [36]. Most studies that have proved the isoplethy theory have used small sample sizes far much less than what was used in this study [36]. As such unequal frequencies have been found in many species that have used large sample sizes for example in *Prunus lannesiana* var. *speciosa* [26], *Prunus spinosa* [16] and *Prunus avium* [36]. Due to lack of isoplethy, it was therefore not possible to estimate the total number of alleles.

### Sequence analysis

The lengths of most of the sequences from both introns were similar to those from previously observed *Prunus S*-RNases [25] [37]. Sequences of the first intron of *P*. *africana* ranged from 210 to 550 bp whilst those of second intron lengths ranged from 245 to 3000 bp. However, *P*. *dulcis* sequences from the first intron are longer (up to 1061 bp) than observed for *P*. *africana* and a size not recorded in any other *Prunus* species [37]. For the second intron, *P*. *cerasifera* has alleles up to 3400 bp [38] relative to the 3000 bp alleles observed in *P*. *africana*. These sequence lengths are also comparable to those detected in *Pyrus pyraster*, *P*. *pyrifolia* and *P*. *communis* with average lengths between 334 and 2000 bp, 352 and 1347 bp and 641 and 2217 bp [39] respectively. Nevertheless, interpretation of allele number as obtained solely by molecular techniques is the question of whether different sequences necessarily correspond to different specificities.

### Synonymous and non-synonymous substitutions

Finding genomic regions under selection is a critical step in elucidating adaptability and adaptiveness of traits and history of populations. The overall Ka/Ks ratio for 14 *P*. *africana S-RNases* was 1.18 which is substantially higher than has been found in both the cultivated *P*. *avium* and *P*. *dulcis* as well as other wild *Prunus* species like *P*. *cerasifera*, *P*. *lannesiana* (0.793) [26], and *P*. *tenella* (0.99) [40]. Although these wild species have consistently had a higher Ka/Ks, this has always been <1, indicating negative selection, which is typical of *S*-alleles that are always under negative frequency dependent selection. Such selection allows rare alleles in a population to increase relative to more common ones by allowing rare alleles to get more compatible mate advantage and also favouring immigrant alleles. Observations made on single populations indicated that the structure of *S*-alleles is less dependent on mutation rate [30]. In contrast, *P*. *africana* Ka/Ks > 1 implying a positive selection for advantageous new mutations in the population. Ecological factors that trigger a great number of adaptive amino acid changes on the *S*-locus have not yet been explored although drastic reduction in effective population size that *P*. *africana* has undergone through logging and habitat degradation can modify the intensity of genetic drift and thus the efficiency of selection against deleterious mutations. This certainly results in a relative excess of non-synonymous changes.

### Phylogenetic analysis

Population size should be revealed not only by number of alleles, but also by divergence time. The *S*-RNases in *Prunus africana* vis a vis other *Prunus* species have shown higher intrasubfamilial similarities. These results corroborate the findings of previous studies [5], that suggest that the *S*-locus of *Prunus* species diverged more recently or evolved at a slower rate, and thus retains a relatively conserved structure. A comparison of the *S*-locus region of the Sc and Sd-haplotypes in almond (*Prunus dulcis*) by Southern blot analysis found that outside of the divergent genomic region around the Sc-RNase gene (≈ 70 kb), the genomic sequence was highly conserved between the two *S*-haplotypes, suggesting that the ≈ 70 kb region contains the entire complement of *S*-determinant genes. Comparatively, P. *africana* appears to have older *S*-alleles than the *S*-alleles of other *Prunus* species with some alleles not being able to form any clade with other alleles. Probably this may support the “out of Africa hypothesis” for *P*. *africana* just like many other plants and animals.

### *P*. *africana S*-RNase spatial genetic structure

The expected patterns of spatial genetic structure (SGS) at target loci will strongly depend on the type of selection involved. Self-incompatibility (SI) system is a frequency-dependent mechanism that is expected to shape the number, frequency distribution, and spatial distribution of self-incompatibility alleles in natural populations. On the contrary though, it is occasionally indicated that SI in a species tends to eliminate existing spatial genetic structures through its obstruction to self-fertilization and cross-fertilization with close individuals that share the same incompatibility alleles [41]. In *P*. *africana*, a positive spatial structure was observed. A significant spatial genetic structure for the *S*-locus was also observed in *P*. *avium* [3]

In *P*. *africana*, a positive spatial structure over scales of approximately 100 m implies a number of interplaying ecological, demographic and other genetic factors, rather than solely SI. In small populations, such as that of *P*. *africana* in this study, interactions between balancing selection and population structure shape the distribution of *S*-alleles, sometimes in nontrivial ways. Although generally in such populations genetically controlled SI systems may impose significant demographic constraints leading to loss of *S*-allele richness that further suppresses mate availability [42] [43]. Drift has, for example, played an important part in the population history of *P*. *africana* [34]. It has also been suggested that in small populations, there is a common tendency for ecological and demographic factors to override genetic influences [3]. *Prunus africana* populations in Mabira forest were observed to have very limited seed dispersal and survival of seedlings.

All *P*. *africana* trees that were monitored in this study showed a prolific fruit and seed set but seedlings exhibited limited survival and were commonly found under the mother trees. This indicated that either limited seed dispersal or the microenvironment after dispersal doesn’t favour recruitment, with strong implication on the SGS. Reduced reproductive success has also been associated with local genetic effects in *Prunus virginiana* [44]. The population of *P*. *africana* has also recently dramatically declined due to overharvesting of the bark for commercial use in treatment of prostate cancer. When populations decline, genetically controlled SI systems may impose significant demographic constraints leading to loss of *S*-allele richness; further suppressing mate availability [42] [43] with implications to SGS and this can also be verified by the positive values of the Tajiman’s test. Drift has, for example, played an important part in the population history of *P*. *africana* [34].

Besides constrained dispersal and regeneration, the observed SGS for *P*. *africana* may also be attributed to the inefficiency of the SI system at preventing selfing or crossing between relatives. Observations indicate that *P*. *africana* may exhibit mixed mating system especially when clumped [45] as it was observed in the Mabira forest population. Results of the ratio of synonymous and non-synonymous substitutions, Ka/Ks > 1, imply a positive selection for advantageous new mutations in the population and allowing novel *S*-alleles into the system may disrupt functionality of the SI system of the plant [46].

## Conclusions

This study has provided important insights into the SI system of *P*. *africana*. The observed similarity of *P*. *africana S*-alleles to other *Prunus* species strengthens the taxonomic placement of the species within this genus. Although empirical evidence is missing that the observed alleles operate a functional SI system, the high number of alleles detected largely indicate an active SI in *P*. *africana*. Crossing experiments involving individuals with known *S*-genotype are strongly encouraged to finally solve this functionality issue.

The results also have important implications for future breeding purposes. To ensure reproductive success and hence sustainability of the species, genebank establishment and clonal development has to maximally diversify the *S*- alleles. As indicated above, further empirical studies are needed to finally prove SI in *P*. *africana*.

## Supporting Information

S1 Data GPS points, GPS points of tree individuals. (XLS)
